# ASO Author Reflections: Neoadjuvant Chemotherapy Does not Increase Postoperative Complications Compared with Upfront Surgery in Gastric Adenocarcinoma: A Population-Based, Nationwide Study in Finland

**DOI:** 10.1245/s10434-023-14886-2

**Published:** 2024-01-11

**Authors:** Putila Emilia, Olli Helminen, Joonas H. Kauppila, the FINEGO Group

**Affiliations:** 1 Surgery Research Unit, Medical Research Center Oulu, Oulu University Hospital and University of Oulu, Oulu, Finland; 2 Upper Gastrointestinal Surgery, Department of Molecular Medicine and Surgery, Karolinska Institutet and Karolinska University Hospital, Stockholm, Sweden

## Past

No large, population-based studies comparing short-term outcomes and complications between neoadjuvant chemotherapy and upfront surgery in gastric adenocarcinoma exist. Clinical guidelines recommend perioperative chemotherapy for patients with stage ≥ IB resectable gastric cancer.^1^ Gastrectomy associates with frequent complications and high mortality.^2^ It is unknown whether surgical risks in neoadjuvant-treated patients are increased outside the selected clinical trial populations. To this date, there are only few small studies on this topic from Asia. They suggest either no significant difference in postoperative complications or less postoperative complications between neoadjuvant chemotherapy and upfront surgery.^3,4^ Because there are no nationwide studies, large Western studies, or studies using standardized definition of complications, it is important to determine the effect of neoadjuvant treatment on surgical risk in gastric cancer.

## Present

The present study was designed to compare postoperative complication rates after gastric cancer resection in patients receiving neoadjuvant therapy compared with upfront surgery by using standardized definitions and in a population-based nationwide cohort in Finland.^5^ After adjustment for key confounders, neoadjuvant therapy was not associated with increased major postoperative complications, pneumonia, anastomotic complications, wound complications, or other complications, reoperations, or short-term mortality compared with upfront surgery.

## Future

The current evidence suggests that neoadjuvant chemotherapy with platinum-based chemotherapy does not seem to increase the surgical risk of gastrectomy. Future studies should evaluate the effects of newer regimens, such as FLOT, on surgical complications, which may differ between regimens. One should not overlook evaluating immune-oncological treatments, such as nivolumab or pembrolizumab, because they are gaining popularity in the neoadjuvant setting of gastric cancer. Patient performance status may preclude neoadjuvant therapy in many instances. There may be some patient groups in whom neoadjuvant therapies are currently underutilized because of fear of complications. Although the dropout rate during neoadjuvant treatment was not available in this cohort, based on current evidence, the potentially increased surgical risk should not be used as grounds for withholding chemotherapy in otherwise eligible patients.
